# Effect of Elastic Modulus on the Accuracy of the Finite Element Method in Simulating Precision Glass Molding

**DOI:** 10.3390/ma12223788

**Published:** 2019-11-18

**Authors:** Honghui Yao, Keyi Lv, Jiarong Zhang, Han Wang, Xiaozhu Xie, Xiangyou Zhu, Jiannan Deng, Shaomu Zhuo

**Affiliations:** 1 State Key Laboratory of Precision Electronic Manufacturing Technology and Equipment, Guangdong University of Technology, Guangzhou 510006, China; 2 Union Optech (Zhongshan) Co., Ltd., Zhongshan 528437, China

**Keywords:** precision glass molding, elastic modulus, viscoelasticity, accuracy

## Abstract

Precision glass molding is a revolutionary technology for achieving high precision and efficient manufacturing of glass aspheric lenses. The material properties of glass, including elastic modulus and viscosity, are highly dependent on temperature fluctuations. This paper aims to investigate the effect of elastic modulus on the high-temperature viscoelasticity of glass and the accuracy of the finite element simulation of the molding process for glass aspheric lenses. The high-temperature elastic modulus of D-ZK3L glass is experimentally measured and combined with the glass cylinder compression creep curve to calculate the high temperature viscoelasticity of D-ZK3L. Three groups of viscoelastic parameters are obtained. Based on this, the molding process of the molded aspheric lens is simulated by the nonlinear finite element method (FEM). The surface curves of lenses obtained by simulation and theoretical analyses are consistent. The simulation results obtained at different initial elastic modulus values indicate that the elastic modulus has a great influence on the precision of the FEM-based molding process of glass aspheric lenses.

## 1. Introduction

Aspheric lenses can effectively eliminate spherical aberration, coma aberration, astigmatism, and other problems existing in the light path by composition of spherical lens. It can also simplify the optical path and reduce the loss of light energy in the propagation of light, thereby achieving a high-quality image and better optical properties [1]. With the rapid development of optoelectronics and information technology, the demand for high-precision glass aspheric lenses has increased dramatically in many fields, such as in mobile phones, security surveillance, automobiles, laser communication, precision measurement and other optical systems, and so on.

Because of the long production cycle and poor precision stability, grinding and polishing cannot meet the needs of mass production of high-precision aspheric lenses [2]. In the precision glass molding process, optical molds with a nanoscale surface roughness and submicron form, would accurately be applied to perform one-time die-casting of the glass preform in the glass transition temperature range. Thus, it has the advantages of a short product cycle, high output, and more environmental protection. However, the precision glass molding process still faces many technical challenges, and it is difficult to ensure the high-precision and high-efficiency manufacturing of high-quality aspheric lenses.

Currently, the finite element method (FEM) simulation-based study on the precision glass molding process has gradually replaced traditional trial-and-error methods, thereby greatly reducing the times and costs of trials and shortening the development cycle of high-performance lenses. In the early study by Yi et al. [3,4], the glass was treated as a Newtonian, viscous fluid, and the generalized Maxwell model was applied to describe the viscoelasticity of glass under a high temperature. The temperature-dependent Young’s modulus values of BK7 and SK5 glasses were measured by a Brillouin scattering experiment [5]. Zhou et al. [6,7] used three kinds of viscoelastic constitutive models to fit the strain and stress of lenses, and they found that the fitting results of strain using the Burgers model were in good agreement with the experimental results in the creep process. Differently, the most accurate fitting result in the stress relaxation process could be obtained by the Maxwell model. Scott [8], Fotheringham [9], Badrinarayanan [10], and others researched the structural relaxation characteristics of glass. The specific heat capacity *Cp* of glass was measured by a differential scanning calorimeter (DSC), and the structural relaxation parameters of the glass were obtained by fitting with the Tool–Narayanaswamy–Moynihan equation (TNM) [11,12,13]. Ananthasayanam et al. [14] studied the effect of the friction coefficient between the lens and mold on the forming accuracy of the lens during the glass molding process by carrying out ring compression tests using a shear friction model. Zhou et al. [15] combined stress relaxation, structural relaxation, and shear friction models to simulate the molding process. They predicted the geometry and refractive index of the lens based on the residual stress and geometry of the lens, and they compensated the shape of the mold based on the simulation results. Su et al. [16,17] studied the effect of the thermal expansion coefficient on the predicted refractive index changes of the lens. They measured the liquid expansion coefficient of BK7 glass, then simulated the change of the refractive index with different thermal expansion coefficients. The simulation results indicated that the liquid thermal expansion coefficient had a great influence on the predicted refractive index change of the lens and the residual stress distribution. Tao [18] clarified that the annealing process significantly affects the refractive index, residual stress distribution, and shape of the lens. Pallicity et al. [19] used the photoelasticity of the glass to measure the residual birefringence of the formed lens by a six-step phase shift method after considering the stress relaxation, structural relaxation characteristics, and friction characteristics. The experimental results were obtained at different cooling rates. Liu et al. [20] optimized the process parameters by combining numerical optimization and simulation analyses. The residual stress of the lens was controlled by optimizing the thermal history parameters in the cooling stage, and the change of lens profile was compensated by optimizing the mold surface. After simulation analysis using the optimized parameters, the residual stress was controlled within 2 MPa, and the form error was controlled within 1 μm.

The material properties of glass, including the elastic modulus, are highly dependent on temperature. However, in the calculation of glass stress relaxation, past literature has mainly adopted the relationship between stress relaxation and elastic modulus at room temperature to describe the glass properties. The effect of high temperatures inducing change in the elastic modulus on the accuracy of the FEM molding process simulation is still ignored. In this paper, the elastic modulus test and cylinder compression test of glass materials at high temperatures are carried out to calculate the stress relaxation parameters under different elastic modulus values. The FEM-based results reveal the influence of the elastic modulus on the internal residual stress of the lens and the prediction accuracy of the profile error at different temperatures.

## 2. Theoretical Models and Method

According to the viscoelastic theory, the stress σ(t) and the strain ε(t) are, respectively, expressed by the following convolution integrals:

(1) $$\sigma \left(t\right)=\int_{0}^{t}E\left(t-\xi \right)\dot{\varepsilon }\left(\xi \right)d\xi +E\left(t\right)\varepsilon \left(0\right),$$

(2) $$\varepsilon \left(t\right)=\int_{0}^{t}J\left(t-\xi \right)\dot{\sigma }\left(\xi \right)d\xi +J\left(t\right)\sigma \left(0\right),$$

where *t* is the time, and $E\left(t\right)=\sigma \left(t\right)/\varepsilon_{0}$ is the time-dependent stress relaxation modulus at a constant strain $\varepsilon_{0}$. $J\left(t\right)=\varepsilon \left(t\right)/\sigma_{0}$ is the time-dependent creep compliance at a constant stress $\sigma_{0}$.

The complex convolutional form can be transformed into a simple algebraic operation by Laplace transform, so the Laplace transform of the above formula can be obtained as follows:

(3) $$\bar{\sigma }\left(s\right)=s\bar{E}\left(s\right)\bar{\varepsilon }\left(s\right),$$

(4) $$\bar{\varepsilon }\left(s\right)=s\bar{J}\left(s\right)\bar{\sigma }\left(s\right),$$

The simplified stress relaxation modulus function $\bar{E}\left(s\right)$ has the following relationship with the creep compliance function $\bar{J}\left(s\right)$:

(5) $$\bar{E}\left(s\right)=\frac{1}{S^{2}\bar{J}\left(s\right)}$$

Equation (5) shows that the stress relaxation modulus $\bar{E}\left(s\right)$ can be obtained by creep compliance $\bar{J}\left(s\right)$, and then *E(*t*)* is obtained by the inverse Laplace transform.

### 2.1. Calculation of Creep Compliance

A constant axial load *F* is applied to the lower mold, stress σ(t)=F/A, where A is the cross-sectional area of the sample, and it can be treated as a constant value. Hence, the creep compliance can be calculated by the equation (t)=ε(t)/σ(t). In the cylindrical compression test, since the cross-sectional area of A of the glass cylinder continuously increases as the compression increases, the axial stress is continuously reduced, so the creep compliance cannot be directly calculated according to the definition.

**Figure 1:**
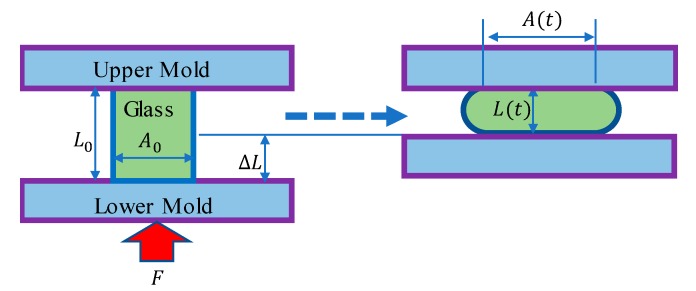
Illustration of creep compression.

By discretizing the creep compliance $J_{i}$ in time, the true strain $\varepsilon_{i}$ and the true stress $\sigma_{i}$ meet the following relationship:

(6) $$J_{i}=\frac{\varepsilon_{i}}{\sigma_{0}}-\frac{1}{\sigma_{0}}\overset{i}{\underset{k=1}{\sum }}J_{i-k}\left(\frac{\sigma_{k}-\sigma_{k-1}}{\Delta t}\right)\Delta t,$$

where $\varepsilon_{i}$ is the true stress of glass. The strain $\varepsilon \left(t\right)=ln\left[\left(l\left(t\right)/l_{0}\right)\right]$; l(t) represents the difference between the initial length of the sample and the length change, $l\left(t\right)=l_{0}-\Delta l$. Then, the true stress $\sigma \left(t\right)=\frac{F}{A\left(t\right)}=\sigma_{0}exp\left[\varepsilon \left(t\right)\right]$. The illustration of parameters is shown in Figure 1.

**Figure 2:**
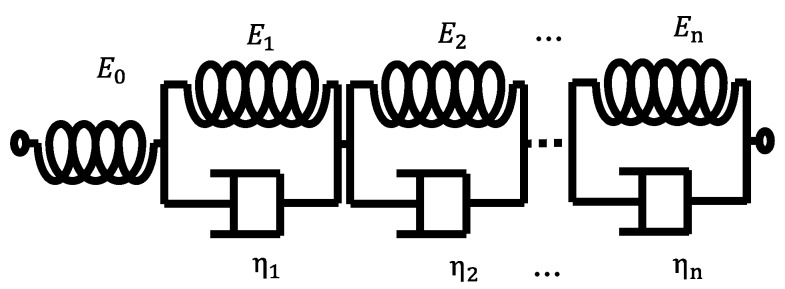
Kelvin–Voigt model.

The creep compliance function is usually characterized by the Kelvin–Voigt model, in which a series of springs and damper units are connected in parallel and connected in series, as shown in Figure 2. The function expression is as follows:

(7) $$J\left(t\right)=\frac{1}{E_{0}}-\overset{n}{\underset{i=1}{\sum }}\frac{1}{E_{i}}\left(1-\exp\left(-\frac{t}{\lambda_{i}}\right)\right),$$

where *E_0_* is the initial elastic modulus, *E_i_* is the elastic relaxation constant of the spring unit *I*, $\lambda_{i}$ is the stress relaxation time in the Kelvin–Voigt model, $\eta_{i}$ is viscosity, and $\lambda_{i}=\eta_{i}/E_{i}$.

### 2.2. Calculation of Shear Relaxation Modulus

The Laplace transform of the creep compliance formula can be written as follows:

(8) $$\bar{J}\left(s\right)=\frac{1}{sE_{0}}+\overset{n}{\underset{i=1}{\sum }}\frac{1}{sE_{i}\left(1+s\lambda_{i}\right)}.$$

**Figure 3:**
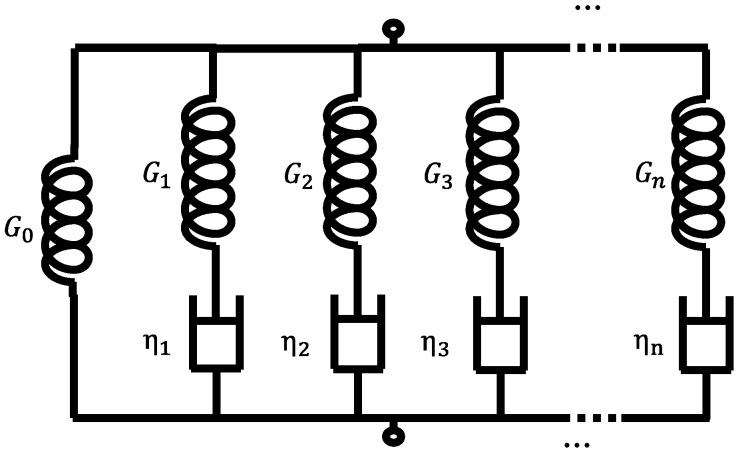
Generalized Maxwell model.

The Laplace transform of the stress relaxation modulus $\bar{E}\left(s\right)$ can be obtained according to Equation (5), and the stress relaxation modulus *E*(*t*) in the time domain is obtained by the inverse Laplace transform. In this case, the Poisson’s ratio *μ* is known, and the shear stress relaxation modulus can be obtained by *G*(*t*) *= E*(*t*)*/[2*(*1 + μ*)*]*. Generalized Maxwell is the most commonly used mechanical analogy model for characterizing the glass stress relaxation behavior. It consists of a series of springs and damping elements connected in series and then in parallel. As shown in Figure 3, the shear stress relaxation modulus is expressed as follows:

(9) $$\left(t\right)=G_{\infty }+\overset{n}{\underset{i=1}{\sum }}G_{i}exp\left(-\frac{t}{\tau_{i}}\right)$$

where $G_{\infty }$ is the shear modulus when the time tends to infinity, *G_i_* is the shear relaxation constant of the spring unit, *τ_i_* is the stress relaxation time and satisfies $\tau_{i}=\eta_{i}/G_{i}$, and $\eta_{i}$ is the viscosity of the damping unit.

The shear relaxation modulus calculated by the above process from different creep compliance formulas is shown in Figure 8.

The stress relaxation of viscoelastic materials is heavily dependent on temperature and has a thermal rheology simple (TRS) behavior. The stress relaxation curves at different temperatures can be aligned with the reference curve after translation on the logarithmic time axis. The translation factor is defined as:

(10) $$\alpha_{T}=\frac{\tau \left(T\right)}{\tau \left(T_{R}\right)},$$

where $\tau \left(T_{R}\right)$ is the relaxation time at a selected reference temperature, and the relaxation time *τ(T)* at other temperatures can be obtained by the translation factor *α_T_*. The temperature effect of the translation factor is often described by the William–Landel–Ferry (WLF) equation:

(11) $$\mathrm{lg}\alpha_{T}=\frac{-C_{1}\left(T-T_{R}\right)}{C_{2}+\left(T-T_{R}\right)},$$

where *T_R_* is the selected reference temperature, and *C_1_* as well as *C_2_* are constant values.

### 2.3. Structure Relaxation

When the glass suddenly changes to temperature *T*_2_ after reaching a state of equilibrium at a certain temperature *T*_1_, which is in the transition region, the equilibrium state will be broken, then the volume of glass will change and slowly approach a new equilibrium state. This phenomenon is called structural relaxation. The relaxation response function is defined by:

(12) $$M_{v}\left(t\right)=\frac{V\left(T_{2},t\right)-V\left(T_{2},\infty \right)}{V\left(T_{2},0\right)-V\left(T_{2},\infty \right)}=\frac{T_{f}\left(t\right)-T_{2}}{T_{1}-T_{2}}.$$

In Equation (12), $V\left(T_{2},t\right)$ is the volume of glass at temperature $T_{2}$ and time *t*, $V\left(T_{2},\infty \right)$ is the volume of glass at temperature $T_{2}$ and infinite time, $V\left(T_{2},0\right)$ is the initial volume of glass at temperature *T*_2_, and the fictive temperature $T_{f}$ describes the degree of deviation of the internal structure from an equilibrium state. In most cases, the response function *M*(*t*) can be expressed by the Kohlrausch function and rewritten by summing n simple exponential functions:

(13) $$M_{v}\left(t\right)=exp\left[-{\left(\frac{t}{\tau_{v}}\right)}^{\beta }\right]=\overset{n}{\underset{i=1}{\sum }}\omega_{i}\exp\left(-\frac{t}{\tau_{vi}}\right),$$

(14) $$\overset{n}{\underset{i=1}{\sum }}\omega_{i}\approx 1,$$

where *β* is the nonexponential parameter between 0 and 1, $\tau_{v}$ is the structure relaxation time, and $\omega_{i}$ is the weight of the structural subrelaxation time $\tau_{vi}$. In this way, some $\tau_{vi}$ values are smaller than $\tau_{v}$, which models experimental cases of the fast kinetics of relaxation functions for short time periods, and some $\tau_{vi}$ values are larger than $\tau_{v},\;$explaining the slow kinetics of the relaxation for long time periods. The TNM model is commonly used to describe the structural relaxation process, where the structural relaxation time $\tau_{v}$ is defined as a function of temperature *T* and fictive temperature $T_{f}$:

(15) $$\tau_{v}=\tau_{v,ref}exp\left\{\frac{\Delta H}{R}\left[-\frac{1}{T_{ref}}+\frac{x}{T}+\frac{\left(1-x\right)}{T_{f}}\right]\right\},$$

where *τ_v,ref_* is the structure relaxation time at the selected reference temperature *T_ref_*; ΔH/R is the ratio of activation energy to ideal gas constant, and *x* is a nonlinear parameter that satisfies 0 < *x* < 1.

## 3. Experiment and Simulation

### 3.1. Composition Analysis of D-ZK3L Glass

**Figure 4:**
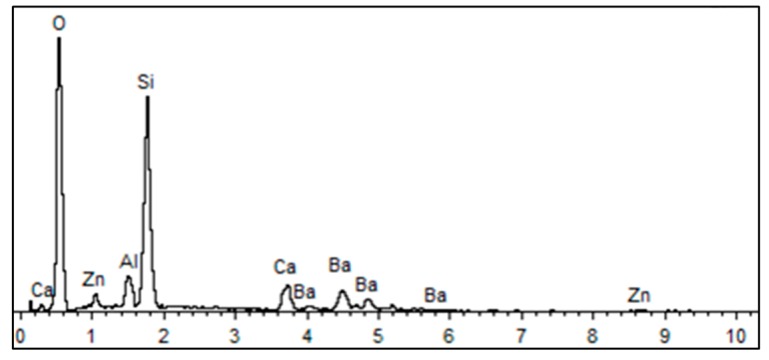
Energy-dispersive X-ray (EDX) spectrum of D-ZK3L.

The optical glass for precision glass molding had a low softening point. In this research, the glass D-ZK3L produced by CDGM (CDGM Glass CO., LTD, Chengdu, China) was used in experiments and simulations. The element composition and energy-dispersive X-ray (EDX) spectrum of D-ZK3L were measured by an electron microscope SU3800 (HITACHI, Tokyo, Japan), in an area of 0.4 × 0.4 mm, and are shown in Table 1 and Figure 4.

### 3.2. Elastic Modulus Measurements of Glass Material

**Figure 5:**
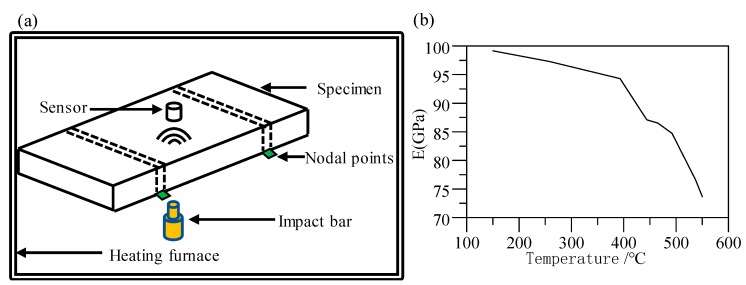
Measurement of elastic modulus: (**a**) illustration of the measurement setup; (**b**) diagram of the elastic modulus of D-ZK3L at high temperatures.

Using the measuring device GrindoSonic^®^ MK7 (GrindoSonic^®^, Leuven, Belgium) [21], the high-temperature elastic modulus of the glass material D-ZK3L between 150 and 550 °C is measured by vibration pulse excitation according to ASTM-1259 [22], and a diagram of the measurement is shown in Figure 5a. During the measurement, the test sample was struck to excite the vibration. Then, the vibration frequency information is collected by the vibration or acoustic sensor. The elastic modulus *E* of the sample is calculated according to the following formula [23,24]:

(16) $$E=0.9465\left(Mf_{f}^{2}/b\right)\left(L^{3}/d^{3}\right)T_{1}$$

where *M* is the sample quality; *b* is the sample width; *L* is the sample length; *d* is the sample thickness; *f_f_* is the vibration frequency; and *T_1_* is the basic bending mode correction factor after considering the sample thickness:

(17) $$T_{1}=1+6.585\left(1+0.0752\mu +0.8109\mu^{2}\right){\left(d/L\right)}^{4}\;\;-\left[\frac{8.34\left(1+0.2023\mu +2.173\mu^{2}\right){\left(d/L\right)}^{4}}{1+6.338\left(1+0.1408\mu +1.536\mu^{2}\right){\left(d/L\right)}^{4}}\right]$$

where *μ* is Poisson’s ratio. The measured results of the elastic modulus of D-ZK3L glass are shown in Figure 5b.

### 3.3. Cylinder Compression Experiment

**Figure 6:**
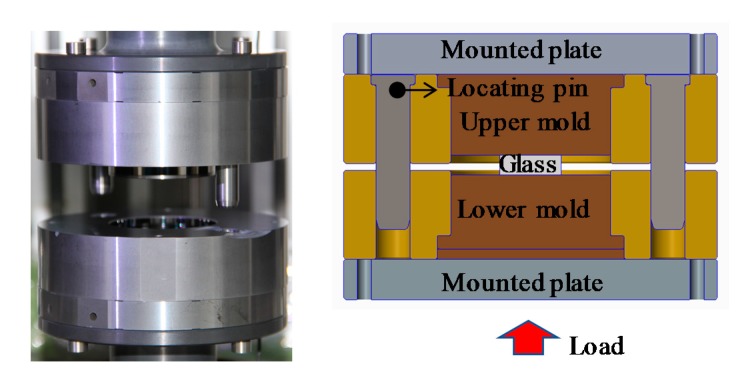
Setup of glass molding.

**Figure 7:**
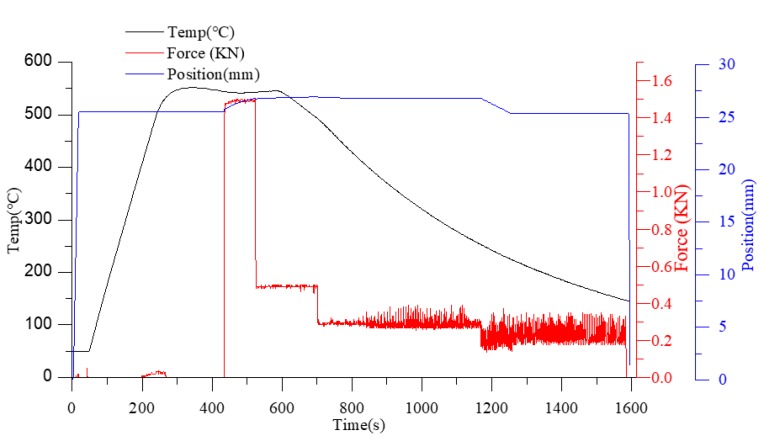
Process parameters for glass molding.

**Figure 8:**
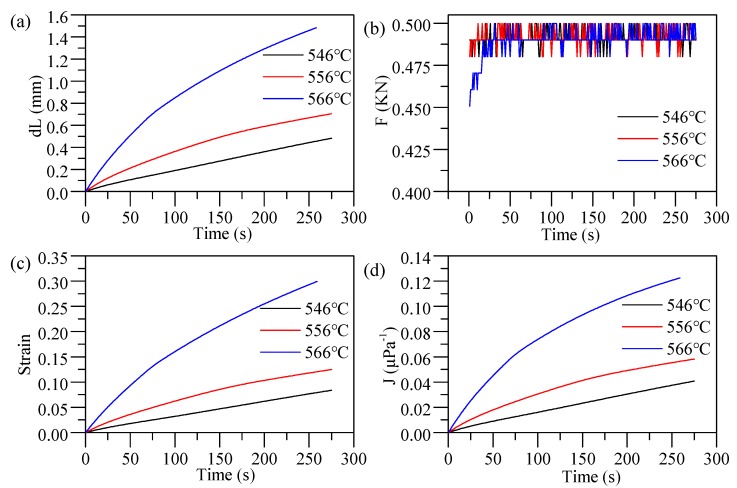
Calculated values for different temperatures: (**a**) thickness variations of the lens; (**b**) load; (**c**) strain; (**d**) creep compliance.

Cylinder compression experiments were performed on cylindrical glass samples (D-ZK3L, φ 18 × 6 mm) with a molding machine GMP-211V (Toshiba Machine, Numazu, Japan) and planar tungsten carbide (J05) cores coated with diamond-like (DLC) film. The setup is shown in Figure 6, and the thermal and mechanical properties of the experimental materials are shown in Table 1. After heating to the molding temperature (three different molding temperatures were employed in this experiment: 546, 556, and 566 °C), the glass was soaked for 200 s in order to ensure a uniform temperature distribution. Subsequently, a constant force of 500 N was applied to cause the glass sample to generate a creep behavior. The displacement variation of the lower mold was recorded and could be assumed to be equal to the thickness variation of the lens in the molding stage. At the end of molding, the glass sample and the core were cooled by nitrogen convection. The specific temperature, displacement of the lower mold, and load change at a molding temperature of 546 °C are shown in Figure 7 and Figure 8.

The measured elastic modulus values at three temperatures were selected as the initial elastic modulus E_0_ in the Kelvin–Voigt model to fit the creep compliance curve according to Formulas (6) and (7). Curve fitting was done using the software 1stOpt (7D-Soft, Beijing, China), and the numerical fitting results and root-mean-square error (RMSE) are shown in Table 1. This indicates that, when n = 3, the RMSE of the fitted curve is small enough. Therefore, the third-order Prony series was selected for calculation.

The reference temperature *T_R_* was selected as 556 °C, and the parameters of the WLF equation were solved by the shear relaxation modulus curve in Figure 8 according to Formula (11). The stress relaxation and the TRS behaviors of D-ZK3L glass at the reference temperature *T_R_* = 556 °C are shown in Table 2.

The parameters of the TNM model were calculated by measuring the specific heat capacity Cp of the glass in a uniform heating process with a differential scanning calorimeter DSC 204F1 (NETZSCH, Selb, Germany). The details of the calculation process are described in reference [14], and the calculation results are shown in Table 3:

### 3.4. FEM Simulation of the Cylinder Compression Process

**Figure 9:**
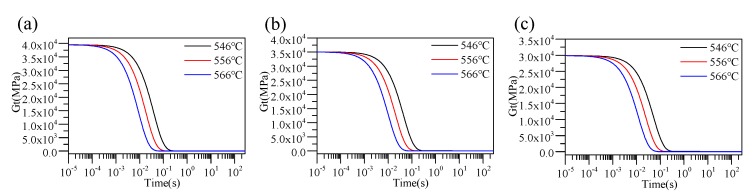
Diagram of the shear relaxation: (**a**) Test 1 (E_0_ = 97450 MPa); (**b**) Test 2 (E_0_ = 86510 MPa); (**c**) Test 3 (E_0_ = 73670 MPa).

**Figure 10:**
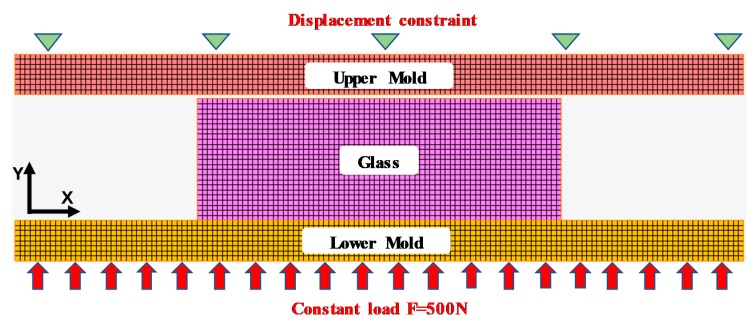
2D finite element method (FEM) simulation model of the glass cylinder compress.

A two-dimensional FEM model of the precise glass molding process was established using nonlinear FEM simulation software, MSC.MARC, as shown in Figure 9 and Figure 10. The molds were set as an elastic body, and the glass cylinder was set as a viscoelastic body. Then, the upper mold was subjected to a displacement constraint, and the lower mold surface was subjected to a force boundary condition along the positive direction of the *y*-axis. Since the sample was soaked for 200 s before molding, the temperature distribution in the sample was assumed to be uniform. Therefore, the glass and the molds were set to a uniform temperature distribution in the FEM simulation model. The characteristic parameters of the material used in the simulations are shown in Table 2, Table 3, Table 4 and Table 5.

**Figure 11:**
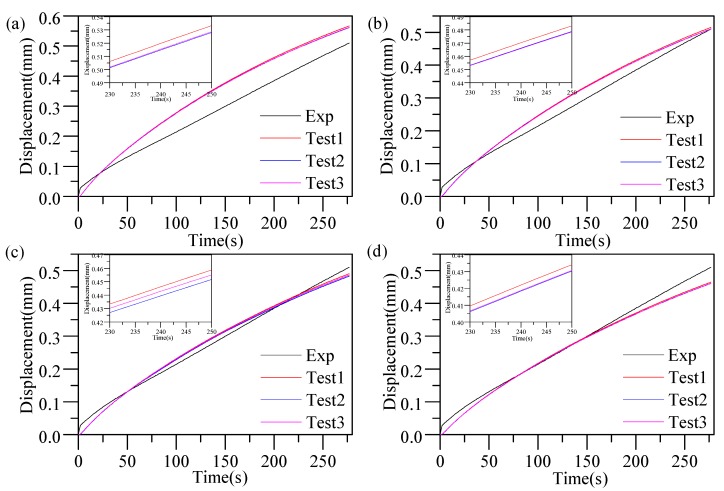
Compression displacement contrast between the simulation and experiment: (**a**) 546 °C; (**b**) 544 °C; (**c**) 543 °C; (**d**) 542 °C.

**Figure 12:**
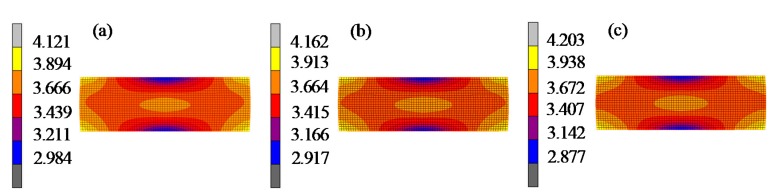
Residual stress contrast of the glass cylinder at 544 °C: (**a**) test 1; (**b**) test 2; (**c**) test 3.

Figure 11 compares the thickness variations of the lens from the simulation and the experiment in GMP-211V. The residual stress distribution of the glass cylinders is shown in Figure 12. The experimental results show that the thickness variation curve of the lens also changed when the elastic modulus at different temperatures was selected as the initial elastic modulus E_0_ for the viscoelastic calculation. The predicted value of the lens thickness variation in Test 1 was the largest. However, compared to tests 2 and 3, the temperature difference was not large enough, and the predicted values were very close. At the same time, the residual stress prediction value of test 1 was the smallest, and test 3 was the largest, as shown in Figure 12.

### 3.5. FEM Simulation of the Glass Aspheric Lens Molding Process

**Figure 13:**
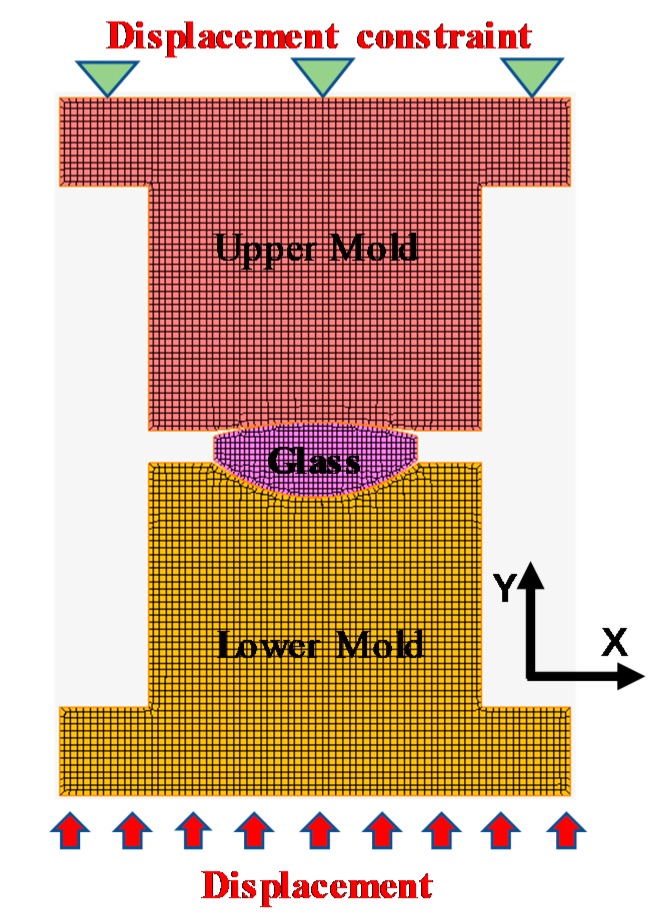
The simulation model of glass aspheric lens molding.

**Figure 14:**
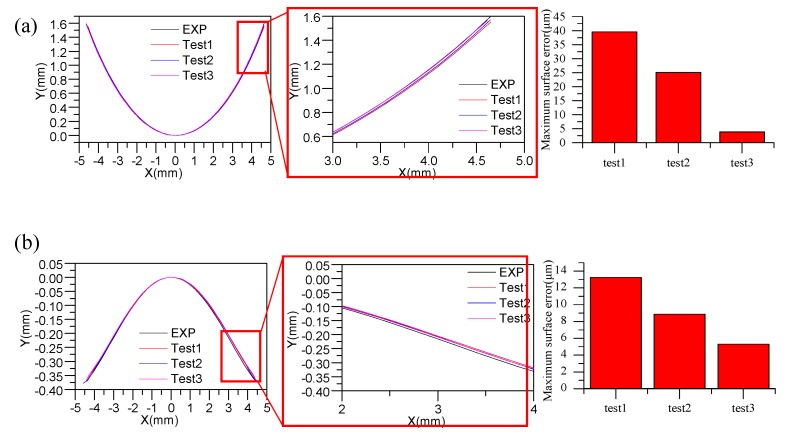
Surface profile contrast between the simulation and experiment: (**a**) surface 1; (**b**) surface 2.

The FEM model for the aspheric lens molding process is shown in Figure 13. The viscoelastic parameters derived from different elasticity modulus values at 25 °C (Test 1), 465 °C (Test 2), and 550 °C (Test 3) were used to simulate the stress–strain response of the glass at a high temperature. The molding temperature was 546 °C, and the soaking time was 200 s. The preform was compressed by applying a load on the lower mold. After the cooling step, a simulated surface profile of the molded lens was extracted and compared with the theoretical surface curve of the lens. The result is shown in Figure 14. The simulation result was close to the theoretical result. Further, test 3 showed minimum form errors on both surfaces.

**Figure 15:**
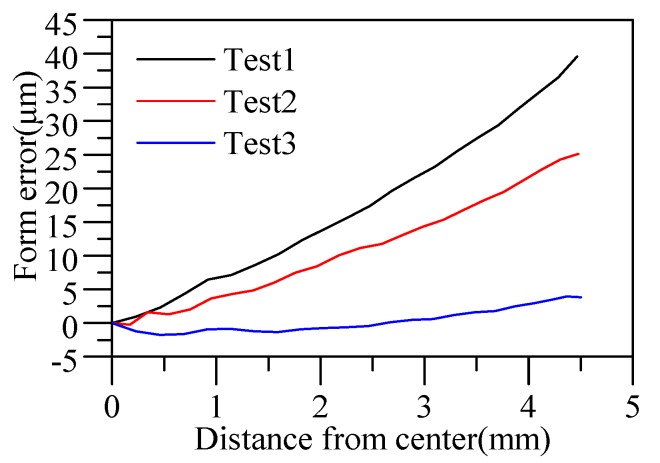
Form errors of simulated surface profiles.

**Figure 16:**
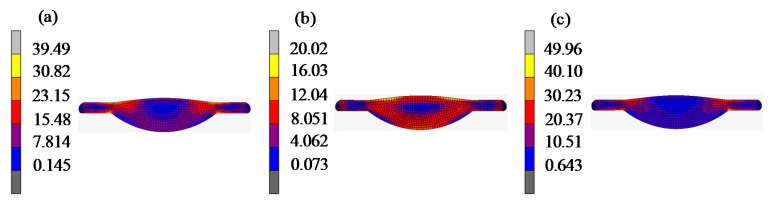
Residual stress lens contrast: (**a**) test 1 (25 °C); (**b**) test 2 (460 °C); (**c**) test 3 (550 °C).

Figure 15 shows the differences between the predicted surface curve and the theoretical surface profile of the lens. Since the lens surface was symmetrical, only the result of the positive *x*-axis is displayed. The results show that the form error of lenses gradually increased from the center to the edge. The predicted surface and the theoretical surface of three groups of different viscoelastic parameters tended to be consistent, indicating that the viscoelastic model obtained by the cylindrical compression creep experiment can characterize the viscoelasticity of D-ZK3L glass at high temperatures. However, by comparing the form error curves of test 1, test 2, and test 3, the predicted errors based on these viscoelastic parameters were different: the form error of test 3 was the smallest, and the form error of test 1 was the largest. The result shows that the closer the initial elastic modulus of the viscoelastic model is to the elastic modulus value at the actual molding temperature, the smaller the prediction error is. At the same time, the residual stresses are also different with different viscoelastic parameters, as shown in Figure 16.

## 4. Conclusions

In this paper, the influence of elastic modulus on the high-temperature viscoelasticity of glass and the accuracy of the FEM in simulating the precision glass molding process were investigated. By measuring the elastic modulus and creep curve of D-ZK3L glass at high temperatures, three groups of different viscoelastic parameters were obtained. Based on the viscoelastic models, an FEM simulation of the precision glass molding process was carried out. The predicted surface shape and the theoretical surface shape of the lens showed good consistencies, indicating the feasibility of calculating the viscoelasticity in the cylindrical creep compression experiment. The conclusions of this study can be drawn as follows.

(1) The elastic modulus declines with the increase in temperatures, from 25 to 550 °C, thereby inducing the change of the viscoelasticity of D-ZK3L glass.
(2) The FEM simulations of the cylinder compression process indicates that the elastic modulus under three different temperatures has a small influence on the compression displacement and residual stress.
(3) The form error of the molded glass aspheric lens can be predicted by FEM simulations of the molding process. A small difference between the initial elastic modulus of the viscoelastic model and the elastic modulus at the actual molding temperature would increase the accuracy of the predicted value.
(4) There is a certain deviation between the simulation and the experimental results in this paper because of the low accuracy of the friction between glass and mold, the real temperature distribution inside the glass and mold, the established viscoelastic model from the molding process and the structure relaxation during the cooling step, as well as the effect on local small scale mechanical properties of glass [25,26]. In future work, the following research topics will be investigated to improve the accuracy of FEM simulations of the precise glass molding process.
  (1) Influence of the friction coefficient between the glass and mold on predicting the lens form error.
  (2) Effect of the temperature distribution of glass on the simulation of precision glass molding.
  (3) Optimization of the calculation method of the structure relaxation model.
  (4) Effect of local, small-size mechanical properties on the prediction of the lens form error.

## Figures and Tables

**Table 1:** Element composition of D-ZK3L glass.

Element Composition	Weight Percentage	Atomic Percent
O K	63.58	80.69
Al K	2.80	2.11
Si K	18.81	13.60
Ca K	3.20	1.62
Zn K	1.68	0.52
Ba L	9.92	1.47
Total	100.00	

**Table 2:** The numerical fitting results of the creep compliance.

Test Temperature (°C)	E_0_ (MPa)	E_1_ (MPa)	E_2_ (MPa)	E_3_ (MPa)	λ_1_ (s)	λ_2_ (s)	λ_3_ (s)	RMSE
25	97450	2.664	1.592	581.230	6905.020	6906.303	14.150	5.31 × 10^−5^
465	86510	1.184	1.875	566.589	9531.571	9531.510	14.600	5.32 × 10^−5^
550	73670	1.465	1.437	567.162	9539.017	9539.131	14.619	5.34 × 10^−5^

**Table 3:** Viscoelastic Parameters.

	Test1 (25 °C)	Test 2 (460 °C)	Test 3 (550 °C)
*E_∞_* (MPa)	0.403	0.294	0.294
*E_1_* (MPa)	39469.438	35,034.378	29827.586
*E_2_* (MPa)	48.708	47.542	47.490
*E_3_* (MPa)	3.269 × 10^−9^	2.811 × 10^−12^	1.049 × 10^−11^
τ_1_(s)	0.038	0.044	0.051
τ_2_(s)	25.975	26.814	26.826
τ_3_(s)	6905.505	9531.535	9539.074
C1	10.224	9.420	9.532
C2	322.624	296.158	299.347
*T_R_* (°C)	556	556	556

**Table 4:** Structural relaxation properties of D-ZK3L.

Structural Relaxation Term	ω_i_	τ_vi_
1	0.026459604	0.937219635
2	0.016604869	2.633717853
3	0.017164272	16.05333599
4	0.144221017	64.99216181
5	0.300269552	814.6405395
6	0.495280686	962.7238416
Activation energy/gas constant, H/R	23099	
Fraction parameter, x	0.46258	
Reference temperature, T_ref_ (°C)	526	

**Table 5:** Thermodynamic characteristics of glass and mold.

Thermo-Mechanical Property	Glass Cylinder (D-ZK3L)	Mold (J05)
Elastic modulus (GPa)	97.45	650
Poisson’s ratio	0.233	0.2
Density (kg/m^3^)	2840	14640
Coefficient of thermal expansion of a solid (K^−1^)	9.81 × 10^−6^	4.6 × 10^−6^
Coefficient of thermal expansion of a liquid (K^−1^)	1.04 × 10^−4^	

## References

[1] Yin S., Jia H., Zhang G., Chen F., Zhu K. (2017). Review of small aspheric glass lens molding technologies. Front. Mech. Eng..

[2] Zhang L., Liu W. (2017). Precision glass molding: Toward an optimal fabrication of optical lenses. Front. Mech. Eng..

[3] Yi A.Y., Jain A. (2005). Compression Molding of Aspherical Glass Lenses-A Combined Experimental and Numerical Analysis. J. Am. Ceram. Soc..

[4] Jain A., Yi A.Y. (2005). Numerical Modeling of Viscoelastic Stress Relaxation during Glass Lens Forming Process. J. Am. Ceram. Soc..

[5] Jain A., Yi A., Xie X., Sooryakumar R. (2006). Finite element modelling of stress relaxation in glass lens moulding using measured, temperature-dependent elastic modulus and viscosity data of glass. Model. Simul. Mater. Sci. Eng..

[6] Zhou T., Yan J., Masuda J., Kuriyagawa T. (2009). Investigation on the viscoelasticity of optical glass in ultraprecision lens molding process. J. Mater. Process. Technol..

[7] Zhou T., Zhou Q., Xie J., Liu X., Wang X., Ruan H. (2018). Elastic-viscoplasticity modeling of the thermo-mechanical behavior of chalcogenide glass for aspheric lens molding. Int. J. Appl. Glass Sci..

[8] Gaylord S., Ananthasayanam B., Tincher B., Petit L., Cox C., Fotheringham U., Joseph P., Richardson K. (2010). Thermal and Structural Property Characterization of Commercially Moldable Glasses. J. Am. Ceram. Soc..

[9] Fotheringham U., Müller R., Erb K., Baltes A., Siebers F., Weiß E., Dudek R. (2007). Evaluation of the calorimetric glass transition of glasses and glass ceramics with respect to structural relaxation and dimensional stability. Thermochim. Acta.

[10] Badrinarayanan P., Zheng W., Li Q., Simon S.L. (2007). The glass transition temperature versus the fictive temperature. J. Non-Cryst. Solids.

[11] Tool A.Q. (1946). Relation between inelastic deformability and thermal expansion of glass in its annealing range. J. Am. Ceram. Soc..

[12] Narayanaswamy O.S. (1971). A Model of Structural Relaxation in Glass. J. Am. Ceram. Soc..

[13] Moynihan C.T., Easteal A.J., De Bolt M.A., Tucker J. (1976). Dependence of the Fictive Temperature of Glass on Cooling Rate. J. Am. Ceram. Soc..

[14] Ananthasayanam B., Joshi D., Stairiker M., Tardiff M., Richardson K.C., Joseph P.F. (2014). High temperature friction characterization for viscoelastic glass contacting a mold. J. Non-Cryst. Solids.

[15] Zhou J., Li M.J., Shen L.G. (2013). FEM Analysis of Glass Lens Molding Press. Adv. Mater. Res..

[16] Su L., Yi A.Y. (2011). Investigation of the effect of coefficient of thermal expansion on prediction of refractive index of thermally formed glass lenses using FEM simulation. J. Non-Cryst. Solids.

[17] Su L., Wang F., He P., Dambon O., Klocke F., Allen Y.Y. (2014). An integrated solution for mold shape modification in precision glass molding to compensate refractive index change and geometric deviation. Opt. Lasers Eng..

[18] Tao B., He P., Shen L., Yi A. (2014). Annealing of Compression Molded Aspherical Glass Lenses. J. Manuf. Sci. Eng..

[19] Dora Pallicity T., Ramesh K., Mahajan P., Vengadesan S. (2016). Numerical Modeling of Cooling Stage of Glass Molding Process Assisted by CFD and Measurement of Residual Birefringence. J. Am. Ceram. Soc..

[20] Liu W., Zhang L. (2017). Numerical optimization platform for precision glass molding by the simplex algorithm. Appl. Opt..

[21] GrindoSonic http://www.grindosonic.com/products/GrindoSonic_MK7.html.

[22] ASTM (1994). Standard Test Method for Dynamic Young’s Modulus, Shear Modulus, and Poisson’s Ratio for Advanced Ceramics by Impulse Excitation of Vibration.

[23] Geng Y., Yan Y., Zhuang Y., Hu Z. (2015). Effects of AFM tip-based direct and vibration assisted scratching methods on nanogrooves fabrication on a polymer resist. Appl. Surf. Sci..

[24] Zhang F., Meng B., Geng Y., Zhang Y., Li Z. (2016). Friction behavior in nanoscratching of reaction bonded silicon carbide ceramic with Berkovich and sphere indenters. Tribol. Int..

[25] Ghidelli M., Gravier S., Blandin J.J., Djemia P., Mompiou F., Abadias G., Raskin J.P., Pardoen T. (2015). Extrinsic mechanical size effects in thin ZrNi metallic glass films. Acta Mater..

[26] Ghidelli M., Idrissi H., Gravier S., Blandin J.J., Raskin J.P., Schryvers D., Pardoen T. (2017). Homogeneous flow and size dependent mechanical behavior in highly ductile Zr65Ni35 metallic glass films. Acta Mater..

